# How a measure of tree structural complexity relates to architectural benefit‐to‐cost ratio, light availability, and growth of trees

**DOI:** 10.1002/ece3.5281

**Published:** 2019-05-26

**Authors:** Dominik Seidel, Peter Annighöfer, Melissa Stiers, Clara Delphine Zemp, Katharina Burkardt, Martin Ehbrecht, Katharina Willim, Holger Kreft, Dirk Hölscher, Christian Ammer

**Affiliations:** ^1^ Silviculture and Forest Ecology of the Temperate Zones, Faculty of Forest Sciences University of Göttingen Göttingen Germany; ^2^ Biodiversity, Macroecology and Biogeography University of Göttingen Göttingen Germany; ^3^ Tropical Silviculture and Forest Ecology University of Göttingen Göttingen Germany

**Keywords:** box‐dimension, fractal analysis, Germany, Indonesia, light availability, plant architecture, productivity

## Abstract

Aboveground tree architecture is neither fully deterministic nor random. It is likely the result of mechanisms that balance static requirements and light‐capturing efficiency. Here, we used terrestrial laser scanning data to investigate the relationship between tree architecture, here addressed using the box‐dimension (*D*_b_), and the architectural benefit‐to‐cost ratio, the light availability, and the growth of trees. We detected a clear relationship between *D*_b_ and the benefit‐to‐cost ratio for the tested three temperate forest tree species (*Fagus sylvatica* L., *Fraxinus excelsior* L., and *Acer pseudoplatanus* L.). In addition, we could also show that *D*_b_ is positively related to the growth performance of several tropical tree species. Finally, we observed a negative relationship between the strength of competition enforced on red oak (*Quercus rubra* L.) trees and their *D*_b. _We therefore argue that *D*_b_ is a meaningful and integrative measure that describes the structural complexity of the aboveground compartments of a plant as well as its relation to structural efficiency (benefit‐to‐cost ratio), productivity, and growing conditions (competition or availability of light).

## 1 INTRODUCTION

Trees are organisms of irregular shape. Among the estimated 3.04 trillion individuals on the planet (Crowther et al., 2015), there is likely no identical pair of trees. From slender, pole‐like, almost branch‐free individuals to extensively growing giants with complex branching patterns, an almost infinite variety of tree shapes occurs across an estimated 60,065 species worldwide (Beech, Rivers, Oldfield, & Smith, 2017). However, tree architecture is not random (e.g., Valladares & Niinemets, 2007). Instead, it is determined by genetical building plans (cf. Hallé and Oldeman 1970) and the myriad of biotic and abiotic factors that act on the tree from the outside. Well documented are the effects of wind (e.g., Noguchi, 1979; Watt, Moore, & McKinlay, 2005; de Langre, 2008), competition strength and type (e.g., Seidel, Leuschner, Müller, & Krause, 2011; Bayer, Seifert, & Pretzsch, 2013; Seidel, Ruzicka, & Puettmann, 2016; Juchheim et al., 2017), water availability (e.g., Archibald & Bond, 2003), light availability (e.g., Kuuluvainen, 1992; Niinemets & Kull, 1995), terrain slope (Barij, Stokes, Bogaard, & Beek, 2007), and other agents that shape trees. The ability of trees to adjust their shape to the environmental conditions, also known as adaptive geometry (Borchert & Slade, 1981; Horn, 1971), is likely a mechanism that balances static requirements (tree stability) and light‐capturing efficiency (Honda & Fisher, 1978; Kuuluvainen, 1992; Valladares & Niinemets, 2007).

In this context, it has been hypothesized that a latitudinal gradient in crown shapes, from flat in the tropics to vertically shaped in higher latitudes, may be the result of an adaptation to the latitudinal gradient in solar inclination angles (cf. Kuuluvainen, 1992; Terborgh, 1985). Latitudinal light gradients are expected “to set different requirements for successful growth strategies of trees,” and it is argued that the trade‐offs between construction and maintenance costs on the one side and photosynthetic gains on the other side are crucial if tree growth and shape are to be understood (cf. Kuuluvainen, 1992). Furthermore, it has been hypothesized that internal crown architecture must enable light penetration in order to provide a selective advantage to trees with a narrow, vertically oriented crown in terms of light utilization (e.g., Kellomäki, Kuuluvainen, & Kurttio, 1986). Advancing our understanding of the above‐mentioned topics has long been complicated, if not made impossible, since adequately characterizing tree architecture with mathematical methods was hardly possible (see Borchert & Slade, 1981).

Here, we argue that fractal analysis can be of great help to gain a better understanding of individual tree shape. This was already suggested by Mandelbrot (1977) and a small number of studies that focused on the issue (e.g., Zeide & Pfeifer, 1991), but only with the recent availability of 3D data on tree architecture, it became possible to exploit the methodology.

The box‐dimension (*D*_b_) seems particularly useful as an integrative measure of tree architecture that is sensitive to both outer shape and internal structure of a tree. Furthermore, it is scale‐independent and hence useful when trees of different sizes are to be compared. Using *D*_b_ has potential to provide a deeper understanding of how structure and function (e.g., growth) are related. Despite its easy calculation (see below), it is not our intention to replace existing and easy‐to‐interpret measures with *D*_b_. Instead, we intend to exploit its potential for scientific use as it may allow understanding why trees and forests shape the way they do. In the following, we will explain why we think so.

**Figure 1:**
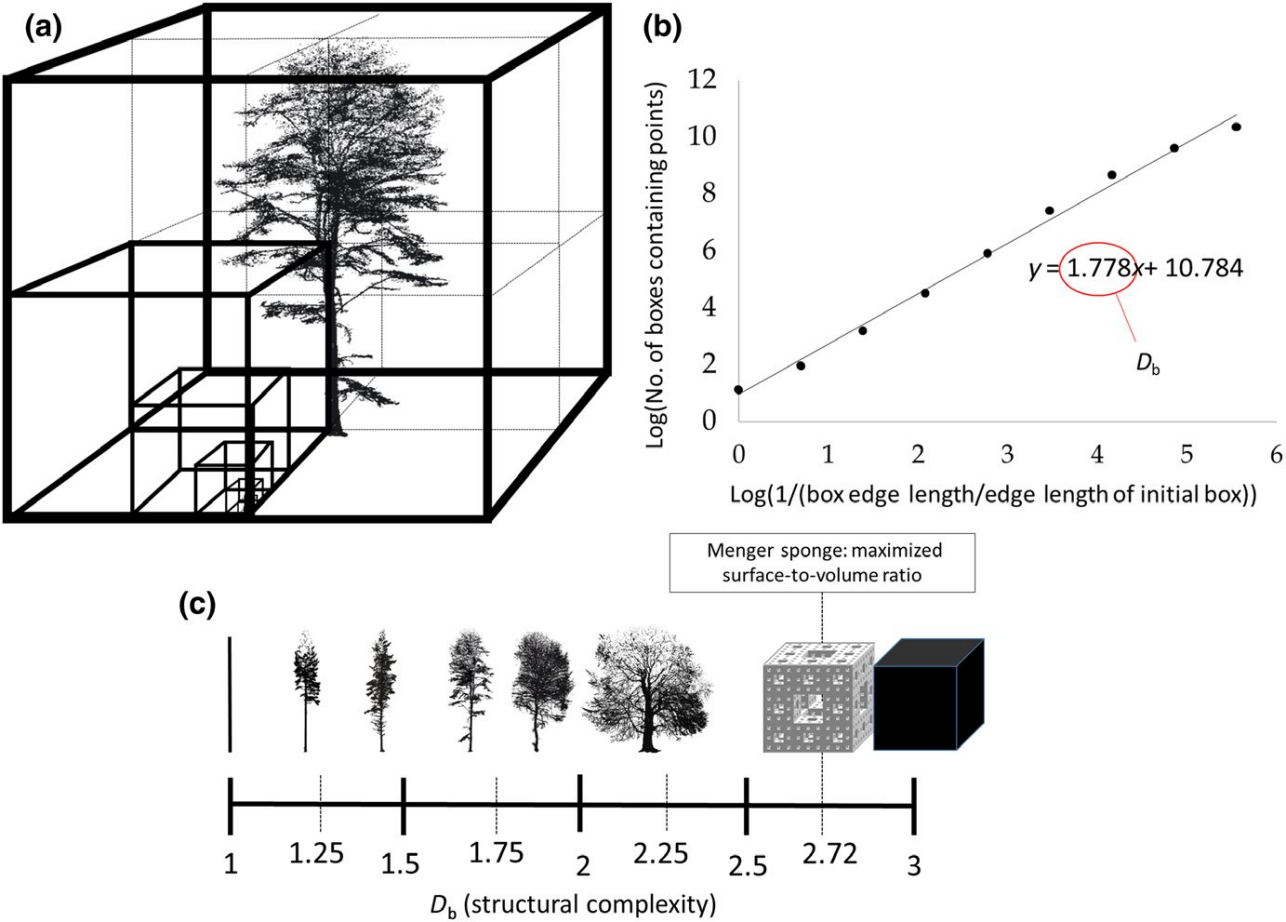
Graphical illustration of the virtual boxes of different sizes the tree point cloud is placed in (a), and the derived linear regression (b) for the natural logarithm of the number of boxes containing points over the inverse of the natural logarithm of the used box size (edge length) relative to the edge length of the initial box. The slope of the regression line equals the box‐dimension of the tree (1.778 in b). Examples of different structural complexities (c) for a cylindrical object (*D*_b_ = 1), across several real‐life tree point clouds (own data, *D*_b_ between 1.18 and 2.2) to the Menger sponge (*D*_b_ = 2.72) and a cube with *D*_b_ = topological dimension = 3

The *D*_b_ of a three‐dimensional (3D) object can be calculated in a straightforward manner if 3D data on the object are available. *D*_b_ is then determined by evaluating how many (virtual) boxes (therefore the term “box‐dimension”; see Figure 1a) one needs to enclose all elements (points) of the 3D tree and how the number of boxes changes with the ratio of the box size to the original box size (largest box encompassing the entire object) used. The slope of the fitted straight line through the scatterplot of the logarithm of the number of boxes needed over the inverse of the logarithm of the used edge length of a box (relative to the initial box size used) is then *D*_b_ (see Figure 1b). Details on the approach can be found in Sarkar and Chaudhuri (1994) and Seidel (2018). It is crucial that for a 3D object like a tree, *D*_b_ can theoretically range from one to three. A *D*_b_ ‐value = 1 is only possible for cylindrical, pole‐like objects (see Figure 1c) that have a diameter smaller than the smallest box size used to measure its *D*_b_. *D*_b_ ‐value = 3 corresponds to solid objects like a cube (see Figure 1c). In this case, *D*_b_ equals the topological dimension of the object (three) as shown in Figure 1c (right end of the scale).

Coming back to trees, we can safely say that only dead, branch‐free trees could approach a *D*_b_ = 1 for their aboveground compartments. On the other end of the spectrum, theoretical considerations that date back far in history (e.g., Leonardo's rule; da Vinci 1452–1519) clearly indicated that a *D*_b_ of three is unrealistic for a tree (cf. also Mandelbrot, 1977). In fact, there are strong indications that a tree's maximum *D*_b_ must be lower than 2.72, which is the *D*_b_ of the Menger sponge (e.g., Alberich‐Bayarri et al., 2010), a mathematical construct developed by Karl Menger (1926), that describes the object with the greatest surface‐to‐volume ratio. Its shape would be the perfect design if a tree would need to maximize its exchange surface to an omnipresent surrounding medium (e.g., air) at minimum building costs and in the absence of competition with other plant individuals. Considering aboveground resources, tree growth is usually not limited by gases in the air, more precisely carbon dioxide, but by the availability of light (e.g., Borchert & Slade, 1981). Accordingly, the need to capture light is much more likely to be the key stimulus for a tree to develop a certain (aboveground) shape than the need to exchange carbon dioxide (cf. Valladares & Niinemets, 2007). Light, as opposed to gas, is not an omnipresent medium “surrounding” a tree, since it comes from a limited range of directions. Hence, to capture light a growth shape similar to a Menger sponge would result in a high level of self‐shading, which would be counterproductive. We therefore assume that maximum *D*_b_ is < 2.72 for the aboveground compartments of trees. *D*_b_ is a measure that may help to understand tree shape and how trees save energy to maintain stability at lowest cost, (e.g., Minamino & Tateno, 2014), that is to maximize light, nutrient and water capture, and accordingly, to increase fitness and growth (cf. Valladares & Niinemets, 2007).

We use 3D data from terrestrial laser scanning to test the hypothesis that (a) the aboveground *D*_b_ is related to the ratio between the crown surface area (a proxy for the photosynthetically active surface of the tree) and the volume of the wooden skeleton of the tree (a proxy for building costs; here, it includes all wooden materials of the tree (stem, branches, twigs)). From here on, we refer to this ratio as “architectural benefit‐to‐cost ratio” of the tree. Furthermore, we hypothesize (b) that *D*_b_ is related to the height growth and diameter increment of trees. We finally hypothesize that (c) *D*_b_ is an integrative measure for the light availability of a tree and it should hence respond to competitive pressure enforced on a tree.

## 2 MATERIALS AND METHODS

### 2.1 Laser scanning

Using the approach presented in Seidel (2018), we determined the aboveground box‐dimension (*D*_b_) of 203 tree individuals belonging to ten different species using Mathematica software (Wolfram Research). We used data from three different scanning campaigns conducted in Indonesia and Germany in order to address the hypotheses stated above. In Section 2.1.1, 2.1.3, a more detailed description of the scanning campaigns is provided. We only present selected analysis for each of the three datasets instead of all analysis for all datasets. This was necessary, as not all data were available for all scanning campaigns.

#### 2.1.1 Box‐dimension versus surface‐to‐volume ratio

**Figure 2:**
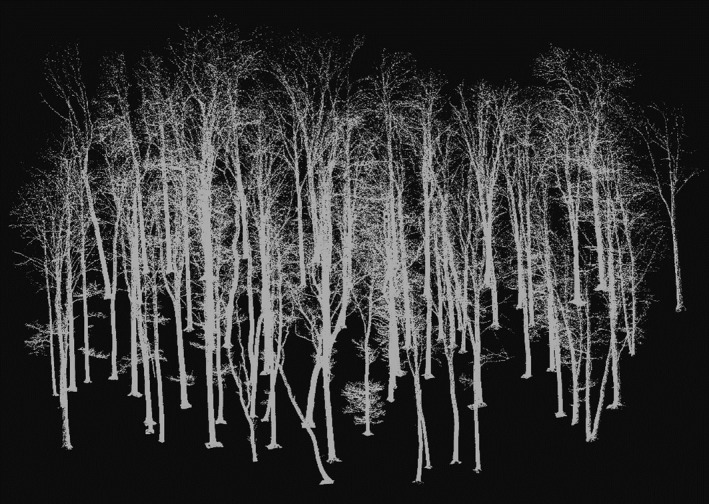
Overview on the laser‐derived point clouds of the 76 selected individuals (beech, maple, and ash) in their natural spatial configuration in the Hainich National Park, Thuringia, Germany

The first dataset consists of 76 individual trees located on a research site in the UNESCO World Heritage Site Hainich National Park (51°4'45.18"N; 10°27'7.62"E; 440 m above sea level) in Thuringia, Germany. Mean annual temperature was 6.8°C in the area (TLWF, 1997), and mean annual precipitation was 872 mm (2000–2002; Knohl, Schulze, Kolle, & Buchmann, 2003). The stand can be characterized as mixed deciduous forest dominated by *Fagus sylvatica* L. (*Hordolymo‐Fagetum*), with an age of around 171 years and around 150 trees/ha (Mund, 2004). It is unmanaged since more than two decades and was previously managed as coppice‐with‐standards forest. The scanning campaign was comprised of 35 laser scans form a Zoller and Fröhlich Imager 5006 3D laser scanner. All scans were made on 5 March 2013 in leafless condition. The 35 scan positions covered an area of 70 m × 70 m. Scans were spatially referenced to each other based on artificial chessboard targets to create a unified point cloud for the entire stand. All trees growing in the area were then manually delineated in 3D and stored as individual point clouds. Based on this, single‐tree information on the woody volume (skeleton volume) for almost 300 trees growing in the scanned area was obtained using Quantitative Structure Models, so‐called QSMs (see, e.g., Hackenberg, Spiecker, Calders, Disney, & Raumonen, 2015; Juchheim et al., 2017; Seidel et al., 2019). We selected a subsample consisting of all trees with a wooden volume greater than 2 m^3^ (in total, 76 individuals, see Figure 2) to exclude trees from the understory. The subsample comprised of the three species *F. sylvatica* L. (beech; *n* = 46), *Fraxinus excelsior* L. (ash; *n* = 25), and *Acer pseudoplatanus* L. (sycamore maple; *n* = 5).

For each tree, we calculated the crown surface area as the convex hull of the point cloud according to Metz, Seidel, Scheffer, Schulze, and Ammer (2013). This measure was used as an estimate of the photosynthetically active surface of the tree. Furthermore, we used the woody skeleton volume as derived from the QSMs (see previous paragraph) as a surrogate for the amount of material “invested” in the structure of the trees. The ratio of the two (crown surface/tree volume) was used as a measure of the benefit‐to‐cost ratio, and we evaluated whether it is related to the *D*_b_ of the individuals.

#### 2.1.2 Box‐dimension versus growth performance

**Figure 3:**
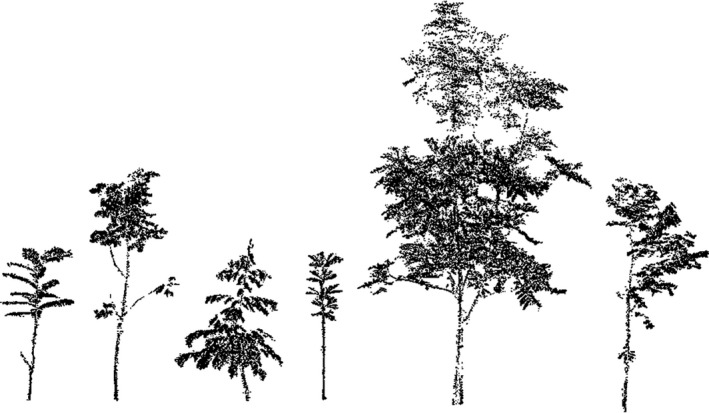
Overview on exemplary individuals (in scale) of the investigated tree species. From left to right: *Parkia speciosa*, *Archidendron pauciflorum*, *Durio zibethinus*, *Dyera polyphylla*, *Peronema canescens*, and *Shorea leprosula*

The second dataset consisted of 40 individuals of six different tropical tree species (see Figure 3), namely *Parkia speciosa* Hassk. (*n* = 6), *Archidendron pauciflorum* (Benth.) I.C. Nielsen (*n* = 8), *Durio zibethinus* L. ex (*n* = 6), *Dyera polyphylla* (Miq.) Steenis (*n* = 8), *Peronema canescens* Jack (*n* = 6), and *Shorea leprosula* (Miq.) (*n* = 6). The trees were scanned in 2017 in leaf‐on condition using a Faro Focus 3D 120 laser scanner (Faro Technologies) and were all growing in a tree planting experiment located in the Jambi Province, Sumatra island, Indonesia (see Teuscher et al., 2016). The trees were scanned from a minimum of four different directions, and scans were spatially referenced using artificial tie points.

In February 2018, average tree height across all species was measured using a measuring tape for trees smaller than 2 m, a telescopic measuring rod for trees between 2 m and 8 m height, and a Vertex (Haglöf) for tree exceeding 8 m in height. A comparison of the height measurement on 20 trees using the vertex and measuring rod indicated 0.6 m = ±3.5% error (Zemp et al., in press). In January and February 2016 and 2017, the diameter was measured at 10 cm above ground (to 0.5 cm accuracy) using calipers. The difference in diameter and height between 2017 and 2016 was used as a measure of tree growth and related to the *D*_b_ of the tree individuals in 2017. Since the trees were small and underwent fast changes in shape in the previous year (visual assessment) due to quick growth, we decided to test whether *D*_b_ explains the growth at temporal proximity to the *D*_b_ measurement (2016–17) instead of growth since planting.

#### 2.1.3 Box‐dimension in dependence of light availability

To test the hypothesis that *D*_b_ is an integrative measure of the light availability a tree is exposed to, we tested how *D*_b_ changed for trees of the same species across a gradient of aboveground competition (as a surrogate for the light availability in the crown), assuming that higher aboveground competition results in lower light availability for the individual. We used a dataset of 93 red oak (*Quercus rubra* L.) trees that were recorded on ten different sites (all dominated by red oak; eight with *n* = 10 trees, one with *n* = 7 trees, and one with *n* = 6 trees) in Germany with varying stem density (min: 22.0 m^2^/ha; mean: 30.9 m^2^/ha; max: 48.1 m^2^/ha). The trees were scanned with four or five scans between January and April 2018 in leafless condition, using a Faro Focus 3D 120 laser scanner (Faro Technologies). We used the Hegyi index (Hegyi, 1974) as a measure of competition enforced on each tree and hence a surrogate for the available light (Metz et al., 2013). For the calculation of the index values of each oak tree, distance to and diameter of all neighboring trees in a radius of 7.5 m (mean crown radius of the study trees was only 2.8 m) that were larger than 7 cm in diameter (at least “pole wood” stage) were measured. Since the ten growth sites differed in environmental conditions (mean annual temperature and precipitation, soil properties, etc.) as well as management history and current management practices, we analyzed the response of *D*_b_ to competition separately for each area.

### 2.2 Statistical analysis

The free statistical software R (Vers.3.4, R Development Core Team) was used for all statistical analysis described in the following.

To analyze the relation between explanatory and response variables, regression models were used. In order to correct for inhomogeneous standard deviations (non‐normally distributed regression residuals, visual verification, Shapiro test), logarithmic transformations were applied for the explanatory and response variable (McDonald, 2014). To correct for the systematic bias as result of the logarithmic transformation, a first‐order correction based on the residual standard error was calculated (Sprugel, 1983). Regression modes were considered significant, if the parameter estimate for the slope resulted in a *p*‐value < 0.05. We used linear regression models to test for possible relationships between *D*_b_ and measures of tree dimension (total tree height (TTH) and DBH). To compare the regression lines between species, we used the species identity as dummy variable to create a design matrix, allowing testing for significant differences between species‐specific intercepts and slopes.

We used analysis of variance (ANOVA) with Tukey's post hoc test (Welch *t* test; *p*‐value: 0.05) to test for differences between the mean *D*_b_ H and *D*_b_ of the species.

## 3 RESULTS

### 3.1 Box‐dimension versus surface‐to‐volume ratio

**Figure 4:**
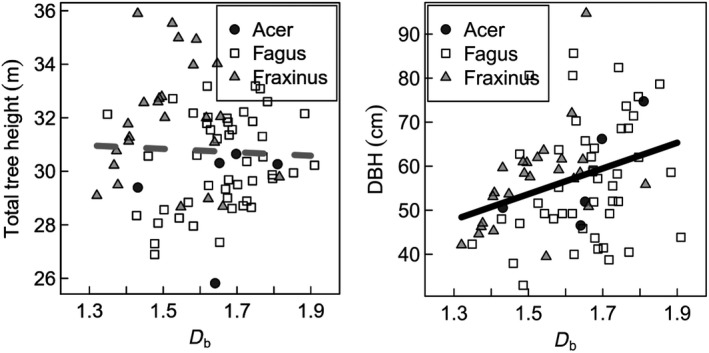
Relationship between tree structural complexity (*D*_b_) and measures of tree dimension. Left: Total tree height, not significant. Right: Diameter at breast height (DBH), significant with *p* = 0.00487 and *R* ^2^ = 0.1022

The results show that the investigated ash trees were significantly larger than beech or maple trees (see Table 1). At the same time, ash trees were of lowest structural complexity (*D*_b_), significantly lower than beech. Testing the relationship between *D*_b_ and measures of tree dimension (total tree height (TTH) and DBH) showed no (TTH) or only a loose (DBH) relationship (*R* ^2^ = 0.1) (Figure 4).

**Table 1:** Overview on tree height (TTH; as a dimensional measure) and box‐dimension (as complexity measure) of the study trees (maple: *n* = 5; beech: *n* = 46; ash: *n* = 25)

Species	Min TTH (m)	Max TTH (m)	Mean TTH (m)	Min *D*_b_	Max *D*_b_	Mean *D*_b_
Maple	25.82	30.65	29.29 a	1.43	1.81	1.65 a,b
Beech	26.89	33.19	30.32 a	1.35	1.91	1.66 b
Ash	28.67	35.90	31.88 b	1.32	1.81	1.52 a

Letters after mean TTH and mean *D*_b_ indicate significant differences in the means between the species (ANOVA, Tukey's post hoc test, *p* < 0.05).

**Figure 5:**
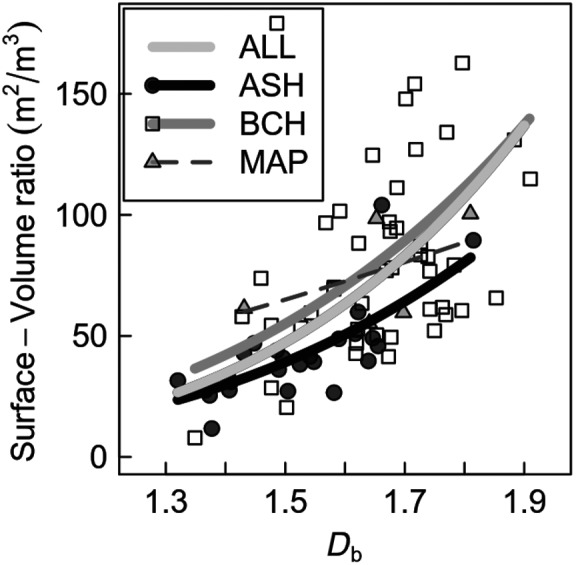
Back‐transformed logarithmic regression models and scatterplot of *D*_b_ against surface‐to‐volume ratio of the trees grouped by species and for all species together. Significant regressions at the level of *p* < 0.05 are bold; All species = ALL, *R* ^2^ = 0.45, *n* = 76; Ash = ASH, *R* ^2^ = 0.53, *n* = 25; Beech = BCH, *R* ^2^ = 0.27, *n* = 45, Maple = MAP, *R* ^2^ = 0.25, *n* = 5. The regression lines (intercept, slope) do not differ significantly between the species

The box‐dimension (*D*_b_) was positively related to the surface‐to‐volume ratio when all trees were considered (Figure 5). A significant relationship was also identified for beech and ash if considered separately, but not for maple at comparable *R* ^2^. This may be explained by the considerably smaller sampling size for maple trees.

### 3.2 Box‐dimension versus growth

**Figure 6:**
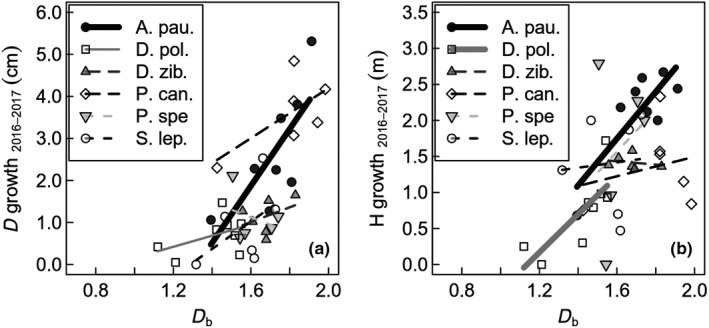
Scatterplots for annual diameter (a) and height (b) growth for the six investigated tropical tree species against the box‐dimension (*D*_b_). Shown are the increments in height (m) and diameter (cm) between 2016 and 2017. Significant regressions at the level of *p* < 0.05 are shown in bold (continuous lines); A. pau. = *Archidendron pauciflorum* (D growth: *R* ^2^ = 0.57; H growth: *R* ^2^ = 0.67); D. pol. = *Dyera polyphylla* (D growth: *R* ^2^ = 0.21; H growth: *R* ^2^ = 0.61); D. zib. = *Durio zibethinus* (D growth: *R* ^2^ = 0.15; H growth: *R* ^2^ = 0.05); P. can. = *Peronema canescens* (D growth: *R* ^2^ = 0.43; H growth: *R* ^2^ = 0.05); P. spe. = *Parkia speciose* (D growth: *R* ^2^ = 0.1; H growth: *R* ^2^ = 0.07); S. lep. = Shorea leprosula (D growth: *R* ^2^ = 0.26; H growth: *R* ^2^ = 0.01)

Average height of the trees was 4.12 m (±2.2 m standard deviation). We found positive relationships between *D*_b_ and the growth expressed as the one‐year increment in diameter (Figure 6a) and height (Figure 6b) for some of the tropical tree species. Even though the slope was only significant for *Archidendron pauciflorum* (diameter, height) and *Dyera polyphylla* (height), the positive trend between *D*_b_ and increment can also be seen for most other species (Figure 6).

### 3.3 Box‐dimension in dependence of light availability

**Figure 7:**
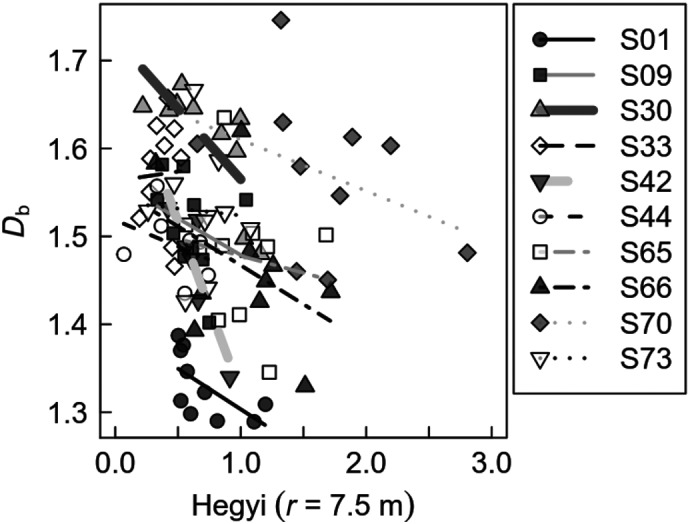
Scatterplots and regression lines for the box‐dimension (*D*_b_) against the competition intensity (Hegyi including trees within a radius of *r* = 7.5 m), by site (S01–S73). Significant regressions at the level of *p* < 0.05 are bold

The *D*_b_ ‐value of tree structure decreased with increasing competition, measured as Hegyi index, and hence decreasing light availability (Figure 7). This trend can be seen on nearly all sites, except for S33. This site, however, only covered a very small gradient in competition (Hegyi from 0.4 to 0.55). Despite the small sampling sizes of all growth sites (*n* = 6–10), the regression models for sites S30 and S42 were significant (*p* < 0.05) and nearly significant for S01 (*p* = 0.05). Only one site showed no relationship between *D*_b_ and the Hegyi index.

## 4 DISCUSSION

Our study showed that, in confirmation of hypothesis (i), a tree's architectural benefit‐to‐cost ratio is positively related to its *D*_b_. The positive relationship between *D*_b_ and a tree's productivity, here observed for tropical tree species and reported earlier for temperate species (Seidel 2018), further indicates that trees benefit from a high *D*_b_, that is, structural complexity. Since *D*_b_ is a dimensionless measure of structural complexity, it is not surprising that for our study trees, which are all adult and dominant or co‐dominant trees reaching the upper canopy layer, it is not (in case of tree height) or only loosely (DBH) related to measures of tree dimension (Figure 4). As our intention was not to find the best predictors for our response variable, but to precisely study the links to complexity, we did not focus on model optimization (best model to predict, i.e., growth) but the analysis of relationships. *D*_b_ is sensitive to the surrounding forest structure a tree is exposed to (growing at gap or in forest interior; see Seidel, 2018), to the competition type (intra‐ or interspecific; cf. Seidel, 2018), and at the same site it differs across species (Seidel, 2018). Furthermore, it is related to tree growth in both temperate (Seidel, 2018) and tropical forests (this study). Branching pattern of trees were also shown to be major drivers of tree structural complexity (Seidel et al., 2019). Summarizing these findings, it is little surprising that we found *D*_b_ to be also sensitive to the competitive strength and the resulting light availability a tree experiences in the present study (confirmation of hypothesis (iii)). As suggested by previous studies (Honda & Fisher, 1978; Kuuluvainen, 1992; Valladares & Niinemets, 2007), the architecture of a given tree is the result of genetic disposition, biomass allocation patterns due to ontogeny, and the abiotic environment in which individuals are assumed to optimize resource capture (e.g., light capturing). From this, we conclude that trees have competitive advantages when they are able to develop a structure that results in a high *D*_b_, or more precisely: The higher a tree's *D*_b_, the more unrestricted was its growth in the past. However, there is a physical limit to this positive relationship. In our study, several trees (tropical and temperate species) reached *D*_b_ ‐values slightly greater than 1.9, but not a single individual crossed the mark of 2, which would still be significantly lower than the *D*_b_ of the Menger sponge (2.72). As mentioned earlier (see Introduction), there seems little use for a tree in achieving the *D*_b_ of the Menger sponge, since self‐shading will automatically result in high building and maintenance costs for branches in the lower and innermost parts of the crown. Trees growing in the open are well able to reach a *D*_b_ greater than 2 (unpublished data), but there is good reason to assume that 2.72 is not only disadvantageous because of the aforementioned reasons, but also physically impossible with respect to the growing pattern of trees and other restrictions, such as hydraulic conductivity. From our data, we cannot answer the question what a *maximum* aboveground *D*_b_ of a natural tree would be, but we can safely say that the *optimal* *D*_b_ depends on the specific tree's growing conditions in the past and present and that it must always be lower than 2.72.

Archibald and Bond (2003) argued that “it is at the whole plant level that the conflicting requirements of various plant functions […] need to be integrated […].” All trees in our study experienced at least some competition, and certainly, none of the trees we investigated grew under complete absence of other stressors (e.g., wind) that could potentially alter the growth pattern. Only under controlled conditions (no wind, no competition, etc.), it is likely that trees develop a genetically predefined structure. This structure is the consequence of an evolutionary adaptation and depends on the tree's reproduction strategy, defense strategy, and other physiological mechanisms (cf. Archibald & Bond, 2003). To a large degree, this structure will also be determined by the evolutionary adaptation to the radiation pattern at a given latitude, from umbrella‐shaped trees in the tropics, over more cylindrical shapes in the temperate zone to cone‐shaped types in the boreal (Kuuluvainen, 1992; Richards, 1957; Terborgh, 1985). While there may be a single optimal shape for a given latitude if light capturing was the only factor to be optimized, other functions need to be optimized as well, such as stability. In combination with genetic determinism and phylogenetic conservatism (Hallé, 1978; Hallé, Oldeman, & Tomlinson, 1978; Tomlinson, 1983), other external drivers of tree shape, such as slope (e.g., Harker, 1996), predominant wind direction (e.g., Brüchert & Gardiner, 2006), herbivory (e.g., Gowda, 1996), fires (e.g., Maze, 2001), will add their part to the final shape. Competition, as investigated here and in earlier studies (e.g., Bayer et al., 2013, Juchheim et al., 2017), further increases the number of processes to be accounted for during architectural development, as the majority of trees worldwide grow in stands rather than solitarily.

The major challenges in applying fractal analysis to tree point clouds seem not in the methodology anymore. Scanning of individual trees is straightforward and will be even more efficient considering the recent developments in the field of handheld laser scanning. This includes trees of all species and in all geographical settings. The methodology is however limited to trees that can actually be extracted from the point cloud. This can be challenging in dense tropical environments or whenever visibility is low or trees grow strongly entangled.

The greater challenge is to decipher the different drivers of complexity and to explain how they act mechanistically. The integrating character of *D*_b_, summarizing the structural complexity into a single number, makes it difficult to gain further detail on specific structural elements responsible for a change in complexity. Future work will focus on the mechanisms behind the complexity–function links.

## 5 CONCLUSIONS

We detected a clear relationship between *D*_b_ and the benefit‐to‐cost ratio of trees, here approximated from terrestrial laser scanning data using the ratio between the crown surface area and the volume of the woody skeleton. In addition, using six tropical tree species, we could also show that *D*_b_ is positively related to the growth performance of a tree, confirming existing findings for temperate tree species. Finally, we observed a negative relationship between the strength of competition enforced on a tree (proxy for light availability) and its *D*_b. _We therefore argue that our study provides evidence for the box‐dimension (*D*_b_) as a meaningful and integrative measure that describes the structural complexity of the whole plant (here: trees) as well as its relation to structural efficiency (benefit‐to‐cost ratio), productivity, and growing conditions (competition or availability of light). We conclude that *D*_b_ is a powerful and comprehensive descriptor of tree architecture and of great potential use for future studies related to the morphology, anatomy, or architecture of trees in varying surroundings and neighborhoods.

## CONFLICT OF INTEREST

The authors declare that they have no conflict of interest.

## AUTHOR CONTRIBUTION

DS, PA, CDZ, ME, CA, HK, and DH contributed to the conceptualization of the study. DS, CA, DH, and HK contributed to the funding acquisition process.DS, KB, MS, and CDZ contributed to data curation process. DS, PA, and ME designed the methodology. DS, CA, DH, and HK involved in the project administration process. DS and PA designed the software. DS, PA, and ME involved in the visualization process. DS, PA, and ME wrote the original draft. ALL wrote, reviewed, and edited the manuscript.

## DATA ARCHIVING

Data are archived using DataDryad and can be found at https://doi.org/10.5061/dryad.b1r6km8.
